# Hantavirus Pulmonary Syndrome, Central Plateau, Southeastern, and Southern Brazil

**DOI:** 10.3201/eid1504.080289

**Published:** 2009-04

**Authors:** Luiz T.M. Figueiredo, Marcos L. Moreli, Ricardo L.M. de Sousa, Alessandra A. Borges, Glauciane G. de Figueiredo, Alex M. Machado, Ivani Bisordi, Teresa K. Nagasse-Sugahara, Akemi Suzuki, Luiz E. Pereira, Renato P. de Souza, Luiza T.M. de Souza, Carla T. Braconi, Charlotte M. Harsi, Paolo M. de Andrade Zanotto, the Viral Diversity Genetic Network Consortium

**Affiliations:** University of São Paulo School of Medicine, Ribeirão Preto, Brazil (L.T.M. Figueiredo, M.L. Moreli, R.L.M. de Sousa, A.A. Borges, G.G. de Figueiredo, A.M. Machado); Adolfo Lutz Institute, São Paulo, Brazil (I. Bisordi, T.K. Nagasse-Sugahara, A. Suzuki, L.E. Pereira, R.P. de Souza, L.T.M. de Souza); University of São Paulo, São Paulo (C.T. Braconi, C.M. Harsi, P.M. de Andrade Zanotto)

**Keywords:** Hantavirus, Araraquara virus, Juquitiba virus, hantavirus pulmonary syndrome, Brazil, research

## Abstract

Hantavirus pulmonary syndrome (HPS) is an increasing health problem in Brazil because of encroachment of sprawling urban, agricultural, and cattle-raising areas into habitats of subfamily *Sigmodontinae* rodents, which serve as hantavirus reservoirs. From 1993 through June 2007, a total of 884 cases of HPS were reported in Brazil (case-fatality rate 39%). To better understand this emerging disease, we collected 89 human serum samples and 68 rodent lung samples containing antibodies to hantavirus from a 2,500-km-wide area in Brazil. RNA was isolated from human samples and rodent tissues and subjected to reverse transcription–PCR. Partial sequences of nucleocapsid protein and glycoprotein genes from 22 human and 16 rodent sources indicated only Araraquara virus and Juquitiba virus lineages. The case-fatality rate of HPS was higher in the area with Araraquara virus. This virus, which may be the most virulent hantavirus in Brazil, was associated with areas that have had greater anthropogenic changes.

## Abstract 2

This syndrome is an increasing health problem because of human encroachment into habitats of rodent reservoirs.

The genus *Hantavirus* of the family *Bunyaviridae* includes a large number of rodent-borne viruses (roboviruses) that are distributed worldwide. Hantaviruses are 80–120 nm in diameter and have an envelope that contains 3 single-stranded, negative-sense segments of RNA known as small (S), medium (M), and large (L). These segments are circular because of their 5′ and 3′ complementary termini and complex with a nucleocapsid (N) protein to form individual L, M, and S nucleocapsids (*1*). The S segment encodes an N nucleoprotein, the M segment encodes a glycoprotein precursor that is processed into Gn and Gc envelope glycoproteins, and the L segment encodes a viral RNA-dependent RNA polymerase. After the first description of hantavirus pulmonary syndrome (HPS) in the United States in 1993, dozens of hantavirus genotypes have been reported in North and South America. These genotypes have been associated with wild rodent species of the subfamily *Sigmodontinae* and are transmitted to humans mostly by contact with or through aerosols of excreta and secretions of infected rodents (*2*). Nevertheless, human-to-human transmission of hantavirus has been reported in Argentina and Chile (*3*).

In South America, hantaviruses have been reported in Argentina (Andes), Chile (Andes), Uruguay (Andes), Paraguay (Laguna Negra), Bolívia (Laguna Negra and Rio Mamoré), Venezuela (Caño Delgadito), and Brazil (Araraquara and Juquitiba) (*4*). Brazil is a large country (area 8,514,215 km^2^), having a predominantly tropical climate and high biodiversity that includes many important zoonoses. This biodiversity in natural ecosystems supports ≈450 of the 540 known species of *Sigmodontinae* rodents (*5*). Furthermore, a human population of ≈190 million is unevenly distributed, mostly in densely populated urban areas near the Atlantic coast of southeastern and northeastern Brazil (Brazilian Institute of Geography and Statistics, 2006, unpub. data).

HPS is an emerging health problem in Brazil because of the overlap of urban, agriculture, and cattle-raising areas with ecosystems containing several species of *Sigmodontinae* rodents that are reservoirs of hantaviruses. From 1993 through June 2007, a total of 877 HPS cases were reported in Brazil (case-fatality rate 39%): 387 in southern Brazil, 264 in southeastern Brazil, 177 in midwestern Brazil, and 49 in northern and northeastern Brazil.

Five lineages of hantavirus have been associated with most documented HPS cases: Juquitiba virus (JUQV), Araraquara virus (ARAV), Laguna Negra–like virus, Castelo dos Sonhos virus, and Anajatuba virus. HPS cases, especially those reported in northern and northeastern Brazil, were likely caused by other unknown hantaviruses (*6–9*; Brazilian Ministry of Health, 2007, unpub. data). Because of a lack of information about genetic diversity among hantaviruses in Brazil, we analyzed nucleotide sequences of hantaviruses infecting persons who contracted HPS and in *Sigmodontinae* rodents to better characterize genotypes and distribution of hantaviruses that cause HPS in an extensive area of Brazil. This area included the southeastern cerrado (a savanna-like ecosystem), the Central Plateau, and southern regions.

## Materials and Methods

### Study Area

The 2,500-km-wide study area in Brazil included the Central Plateau in the midwestern region, the southeastern and southern regions, and the Central Plateau in Goiás State and the Federal District. The Central Plateau was originally a cerrado characterized by small trees and grasses adapted to climates with long dry periods. However, during the past 3 decades the Central Plateau has been modified by farming, creation of pastures, and extensive urbanization. The southeastern region is the most densely populated region of Brazil and includes the states of São Paulo and Minas Gerais, which have cerrado in western areas and neotropical Atlantic rain forest along the coast. The rain forest is an umbrofilous tropical forest on hillsides and has high precipitation caused by an orographic effect. The southern region has Araucaria forests at higher altitudes and neotropical Atlantic rain forest along the coast. These ecosystems sustain *Sigmodontinae* rodents and have been modified, segmented, and damaged by extensive sugar cane, soybean, and coffee farming; cattle raising; and rapid and poorly planned urbanization.

### Collaborative Structure

Collection and serologic analysis of hantavirus samples from humans and rodents were performed at the Adolfo Lutz Institute in São Paulo and at the Virus Research Unit of the School of Medicine, University of São Paulo in Ribeirão Preto. Initial detection of hantavirus genomic RNA was conducted at the Virus Research Unit of the University of São Paulo. Samples positive for genomic RNA were sent to the Microbiology Department of the Institute of Biomedical Sciences at the University of São Paulo for further PCR amplification, DNA sequencing, and sequence analyses.

### Human and Rodent Samples

Human serum samples obtained from HPS patients during 1998–2005 were analyzed by immunoglobulin (Ig) M capture ELISA by using antigens for Andes virus and ARAV. Rodents were captured alive by using traps in rural or sylvan environments at presumed sites of HPS cases during 2002–2005 as part of routine surveillance procedures conducted by the Adolfo Lutz Institute under the mandate of the Brazilian Ministry of Health. Sampling was reviewed and approved by appropriate ethical committees on human and animal research according to Brazilian Ministry of Health Resolutions 3747/95 and 196/96. Informed consent was obtained from all patients, and information was kept confidential by the Ministry of Health. Rodent collection in the field was authorized by the Brazilian Institute of Environment and Renewable Natural Resources. Samples containing hantavirus are under control of the Ministry of Health at classified sites according to United Nations Security Council Resolution 1540 and Brazilian Ministry of Science and Technology Resolution 10. Rodent blood samples were analyzed by IgG ELISA by using antigens for Sin Nombre virus and ARAV. Rodent lungs and human serum samples positive for hantavirus were used for RNA extraction.

### Extraction of RNA

RNA was extracted from 300 μL of hemolyzed whole blood or serum samples from humans and from 300 μL of a suspension of macerate of lung tissues from rodents. Samples were mixed with 1 mL of TRIzol LS Reagent (GIBCO/BRL, Gaithersburg, MD, USA) and 200 μL of chloroform–isoamyl alcohol (24:1), according to modifications described by Bowen et al. (*10*). Pellets were precipitated by centrifugation and resuspended in 10–20 μL of diethyl pyrocarbonate–treated water.

### Reverse Transcription–PCR Primers, RNA Samples, and Reactions

Highly conserved regions of N and Gn genes of ARAV (GenBank accession nos. AF307325 and AF307327) were used to identify primers after aligning their nucleotide sequences to those of American hantaviruses. Two primer pairs, SAHN (amplifying 264 bp) and HANGn (amplifying 324 bp), were used (Table 1). The 2 sets of primers were used for diagnosis of hantavirus infection and DNA sequencing. Serum samples were considered positive for hantavirus if either N or Gn reverse transcription–PCR (RT-PCR) gave the expected amplicon size. Regions of hantavirus N and Gn genes were detected by RT-PCR in RNA extracts of serum or hemolyzed whole blood from 19 HPS patients and RNA extracts of lung tissues from 15 rodents obtained during 2002–2005. Information on these samples is shown in Table 2.

**Table 1:** Primers used for reverse transcription–PCR of hantaviruses, Brazil, 1998–2007

Gene*/primer	Sequence (5′ → 3′)	Nucleotide annealing site
N/SAHN-C	CAAAACCAGTTGATCAACAGGG	213–236 of hantavirus small RNA segment
N/SAHN-S	GATGAATCATCCTTGAACCTTAT	454–477 of hantavirus small RNA segment
G1/HANGn-C	GGGCAGTAAGTGCTGAAAC	1301–1320 of hantavirus medium RNA segment
G1/HANGn-S	ACATTTAGCAGTTTGCCATGGG	1602–1625 of hantavirus medium RNA segment

*N, nucleocapsid; G1, glycoprotein 1.

**Table 2:** Geographic origin of human and rodent sources of hantaviruses, Brazil, 1999–2005

Composite taxon*	City	State	Region	Amplicon†
PR_DF_Hsp_19	Paranoá	Distrito Federal	Central Plateau	N
SS_DF_Nlas_13	São Sebastião	Distrito Federal	Central Plateau	Gn
SS_DF_Nlas_10	São Sebastião	Distrito Federal	Central Plateau	Gn, N
SS_DF_Nlas_11	São Sebastião	Distrito Federal	Central Plateau	Gn, N
SS_DF_Nlas_12	São Sebastião	Distrito Federal	Central Plateau	Gn, N
CO_GO_Hsp_20	Cocalzinho	Goiás	Central Plateau	Gn
AR_SP_Hsp_21	Araxá	Minas Gerais	Central Plateau	Gn, N
SG_MG_Nlas_8	São Gotardo	Minas Gerais	Central Plateau	Gn, N
SG_MG_Nlas_9	São Gotardo	Minas Gerais	Central Plateau	Gn, N
AG_SP_Ost_1	Aguaí	São Paulo	Southeast	Gn, N
BA_SP_Hsp_2	Batatais	São Paulo	Southeast	Gn, N
BA_SP_Hsp_1	Batatais	São Paulo	Southeast	Gn, N
CJ_SP_Hsp_3	Cajurú	São Paulo	Southeast	Gn, N
CJ_SP_Hsp_4	Cajurú	São Paulo	Southeast	N
CC_SP_Hsp_5	Cássia dos Coqueiros	São Paulo	Southeast	Gn, N
CC_SP_Hsp_6	Cássia dos Coqueiros	São Paulo	Southeast	Gn, N
CC_SP_Nlas_2	Cássia dos Coqueiros	São Paulo	Southeast	Gn, N
CC_SP_Nlas_3	Cássia dos Coqueiros	São Paulo	Southeast	Gn, N
CC_SP_Nlas_4	Cássia dos Coqueiros	São Paulo	Southeast	Gn, N
CC_SP_Nlas_1	Cássia dos Coqueiros	São Paulo	Southeast	Gn, N
CC_SP_Nlas_5	Cássia dos Coqueiros	São Paulo	Southeast	Gn, N
CV_SP_Hsp_7	Cravinhos	São Paulo	Southeast	Gn, N
GU_SP_Hsp_9	Guariba	São Paulo	Southeast	N
GU_SP_Hsp_8	Guariba-SP	São Paulo	Southeast	Gn, N
IB_SP_Nlas_6	Ibaté	São Paulo	Southeast	Gn, N
IB_SP_Nlas_7	Ibaté	São Paulo	Southeast	Gn, N
JD_SP_Hsp_10	Jardinópolis	São Paulo	Southeast	Gn, N
JU_SP_Hsp_11	Jaú	São Paulo	Southeast	Gn, N
PO_SP_Hsp_12	Pontal	São Paulo	Southeast	Gn
RB_SP_Hsp_13	Ribeirão Bonito	São Paulo	Southeast	Gn, N
RP_SP_Ako_1	Ribeirão Preto	São Paulo	Southeast	Gn, N
SC_SP_Hsp_14	São Carlos	São Paulo	Southeast	Gn, N
SE_SP_Hsp_15	Sertãozinho	São Paulo	Southeast	Gn, N
ST_SP_Hsp_17	Sertãozinho	São Paulo	Southeast	Gn, N
ST_SP_Hsp_16	Sertãozinho	São Paulo	Southeast	Gn, N
TB_SP_Hsp_18	Taubaté	São Paulo	Southeast	Gn, N
SE_SC_Oni_1	Seara	Santa Catarina	South	Gn
CX_RS_Hsp_22	AY740623	Rio Grande do Sul	South	Gn, N

*The first 2 letters indicate the city, the next 2 the state, and the next 3 the animal source (Hsp, human; Nlas, *Necromys lasiurus*; Ost, *Oligoryzomys stramineus*; Ako, *Akodon* sp.; Oni, *O*. *nigripis*). Numbers indicate sample number. †N, nucleocapsid; Gn, glycoprotein.

### Reverse Transcription

RT reactions were prepared in a final volume of 22 μL by mixing 5 μL of extracted RNA, 0.113 mmol/L deoxyribonucleoside triphosphates (dNTPs), 0.68 μmol/L of either SAHN-C or HANGn-C primer, and 4.4 μL of 5× RT buffer (250 mmol/L Tris-HCl, pH 8.3, 15 mmol/L MgCl_2_, 50 mmol/L dithiothreitol). Reaction mixtures were heated at 95°C for 3 min to linearize RNA and cooled to 4°C. A total of 10 U RNase inhibitor (Pharmacia, Piscataway, NJ, USA) and 10 U of Moloney murine leukemia virus reverse transcriptase (Pharmacia) were added to each sample, and samples were incubated for 2 h at 37°C. The cDNA was used as a template in the subsequent PCR or stored at –20°C.

### PCR

PCRs were conducted in 50-μL volumes containing 0.3 μM of SAHN or HANGn primer pairs, 0.05 mmol/L dNTPs, 3 μL RT products, and 5 μL 10× buffer (100 mmol/L Tris-HCl, pH 8.5, 500 mmol/L KCl). Mixtures were heated to 80°C, 1U of thermostable Taq DNA polymerase (Pharmacia) was added, and mixtures were subjected to 35 cycles at 95°C for 30 s, 55°C for 45 s, and 72°C for 240 s. Samples were then subjected to an elongation step at 70°C for 5 min to promote DNA strand extension. Eight microliters of amplified DNA was subjected to electrophoresis on 1.7% agarose gels in Tris-acetate-EDTA buffer and stained with ethidium bromide. Amplicons were visualized by using a 312-nm UV transilluminator, and their sizes were determined by comparison with a 100-bp DNA ladder.

### Nucleotide Sequencing

Direct nucleotide sequencing of N (261 bp) and Gn (324 bp) PCR products was performed by using the Big Dye Termination Cycle Sequencing Ready Reaction Kit (Applied Biosystems, Foster City, CA, USA) with 25 cycles at 95°C, 55°C for 45 s, and 72°C for 180 s. Products were analyzed in ABI PRISM 3100 and ABI 377 sequencers (Applied Biosystems). Sequences obtained were deposited in GenBank (accession nos. EU170162–EU170239).

### Phylogenetic Analysis

Nucleotide sequences of N and Gn genes were aligned with those of orthologs from other South American hantaviruses (Table 3) by using ClustalX version 8 software (*11*). Alignments were edited by using SeAl version 2.0a11 software (http://tree.bio.ed.ac.uk/software/seal). Preliminary analysis of N and Gn amplicons and references (Table 2) (concatenated in a 573-member dataset that included 58 taxa for which N and Gn are available) or individual genes by using the RDP3 program (*12*) indicated no intergene recombination or reassortment that would be detected by recombination at the border between N and Gn genes in the 573-bp concatamer. This finding confirmed our previous results (*13*).

**Table 3:** Reference hantavirus gene sequences

Vírus	Country of origin	GenBank accession nos.	
		Glycoprotein sequences	Nucleocapsid sequences
Laguna Negra	Paraguay	AF005728	AF005727
Lechiguanas	Argentina	AF028022	AF482714
Oran	Argentina	AF028024	AF482715
Pergamino	Argentina	A028028	AF482717
Araraquara Lutz	Brazil	AY970821	None
Araraquara Johnson	Brazil	AF307327	AF307325
Andes Chile	Chile	AY228238	AY228237
Andes AH-1	Argentina	AF324901	AF324902
Choclo	Panama	DQ285047	DQ285046
Cano Delgadito	Paraguay	DQ284451	DQ285566
Juquitiba	Brazil	AY963900	EF446280
Rio Mamoré	Bolivia	AY953445	U13455
Araucária HR0271	Brazil	None	AY740624
Araucária HR0150	Brazil	None	AY740622
Araucária HR4101	Brazil	None	AY740632
Araucária HR0273	Brazil	None	AY740626
Araucária HR0272	Brazil	None	AY740625
Araucária HR4102	Brazil	None	AY740633
Araucária HR0399	Brazil	None	AY740630
Araucária HR0395	Brazil	None	AY740628
Araucária HR0397	Brazil	None	AY740629
Araucária HR3100	Brazil	None	AY740631
Araucária HR0285	Brazil	None	AY740627
Araucária HR0155	Brazil	None	AY740623

To improve the phylogenetic signal, we used the 573-bp contamer and inferred the maximum clade credibility (MCC) tree for 58 South American hantaviruses by using BEAST version 1.4.8 software (*14*) and the Bayesian Skyline model under exact conditions as described (*13*). The MCC tree was sampled from 20 million trees after Markov chain Monte Carlo algorithm sampling was stable after a preburning stage of 30 million chains. Seven of 38 taxa had neither N nor Gn sequences (Table 2), but this absence did not preclude use of tree-based Bayesian methods and maximum likelihood methods. These methods account for missing nucleotides as undefined character-states during phylogenetic reconstruction and do not use global pairwise distances. Statistical support for clustering of newly isolated viruses was evaluated by running 500 nonparametric bootstraps with GARLI version 0.95 (*15*) and PhyML version 2.4.5 software (*16*). We also conducted a 5% jackknife resampling analysis of 1,000 neighbor-joining trees by using maximum likelihood distance with PAUP version 4b10 software (*17*). The best evolutionary model was estimated with GARLI software (i.e., general time reversible + Γ + I model).

### Case-Fatality Rate of HPS in Brazil

The Central Plateau and southeastern region of Brazil are covered mostly by cerrado, where *Necromys lasiurus*, the reservoir of ARAV, is enzootic. The HPS case-fatality rate for this area was compared with the HPS case-fatality rate for the southern region, which is characterized by Atlantic rain forest and Araucaria subtropical forest environments. In these environments, *Oligoryzomys nigripes*, a reservoir of JUQV, is enzootic. HPS case-fatality rates for the Central Plateau and southeastern region were compared with HPS case-fatality rates for hantaviruses in other regions of Brazil. These comparisons were made by using the Fisher exact test at a 5% significance level (Brazilian Ministry of Health, 2007, unpub. data).

## Results

### Composite Phylogenetic Tree of N and Gn Genes

We studied 89 human samples and 68 rodent samples that had positive serologic results; N and Gn genes from 22 human and 16 rodent sources were obtained. The N/Gn composite tree (Appendix Figure) indicates that human and rodent samples were associated with ARAV or JUQV reference strains with posterior probabilities >0.9. As expected, ARAV samples from rodents and humans in São Paulo State, the Central Plateau, and Minas Gerais State (on the border of the plateau) are mixed in the tree because human cases are almost always derived from rodents (Appendix Figure). Nevertheless, highly supported clusters of ARAV suggest that viruses may show some geographic partitioning (e.g., clusters of isolates from *N*. *lasiurus* in São Sebastião in the Central Plateau differ from clusters from São Paulo in the southeastern region). Furthermore, 13 of 14 ARAV samples obtained from rodents were from *N*. *lasiurus* and 1 was from *Akodon sp*. All rodent and human ARAV samples were from the Central Plateau (cerrado) or southeastern region (tropical rain forests). Distribution of ARAV extends 1,000 km across the dry northern cerrado region and includes western São Paulo State to the boundary of the Atlantic rain forest in the southeastern region.

ARAV are more related to Pergamino virus than to JUQV (Appendix Figure). Pergamono virus infects *A*. *azarae*, which inhabit Argentina, and JUQV infects *Oligoryzomys* spp., which inhabit southern Araucaria and eastern Atlantic forests. Two rodent samples nested with JUQV (high posterior probability 0.93) 100% of the time during jackknife resampling and 86% of the time during 500 nonparametric bootstrap iterations (Appendix Figure). These samples were identified as AG_SP_Ost_1 isolated from *O*. *stramineus* in Aguaí, São Paulo, and SE_SC_Oni_1 isolated from *O*. *nigripis* in Seara, Santa Catarina State located in southern Brazil near the northern border with Argentina (Table 2). These data indicate that these 2 samples were divergent lineages of JUQV infecting *Oligoryzomys* spp. Detection of AG_SP_Ost_1 in Aguaí, São Paulo, was expected because Itapuã JUQV is also found in the adjacent Atlantic rain forest. Two additional samples, SG_MG_Nlas_8 from *N*. *lasiurus* in São Gotardo, Minas Gerais State (Central Plateau), and CX_RS_Hsp_22 from a human case in Caxias do Sul, Rio Grande do Sul (southern region), appeared to have some distant association with ARAV, JUQV, and Oran hantavirus, which are associated with *O*. *longicaudatus* from subtropical Oran Department in Argentina. This association was indicated by the low support of nodes connecting these 3 virus lineages to SG_MG_Nlas_8 and CX_RS_Hsp_22. Although more data are needed to better understand these associations, the most parsimonious explanation is that a hantavirus associated with *Necromys* spp./*Akodon* spp. that originated in the southern region may have been the source of the lineage that led to *Oligoryzomys* spp.–associated JUQV, which has lineages across the Araucária pine forests and along the rain forests into southeastern Brazil.

### Virulence of Hantaviruses Determined by Case-Fatality Rate

The Central Plateau and southeastern and southern regions contain >80% of the HPS cases reported in Brazil in 2007. However, the case-fatality rate of HPS was distinct in these and other regions. These rates were 44.5% (149 deaths in 335 reported cases) in areas with ARAV in the Central Plateau and the southeastern region and 35.4% (192 deaths in 542 cases) in areas with ARAV in the remaining southern regions. This difference was statistically significant (p = 0.011, by Fisher exact test, and χ^2^ 3.0978, df 1, p<0.1). The case-fatality rate of HPS in the Central Plateau and southeastern regions with ARAV (44.5%) was significantly higher than in southern regions with JUQV (32.5%, 126 deaths in 387 cases) (p = 0.0051, by Fisher exact test, χ^2^ 4.8293, df 1, p<0.05) (Brazilian Ministry of Health, 2007, unpub. data). Although some geographic overlap of ARAV and JUQV occurs, these results suggest that ARAV strains may have higher virulence than JUQV or other hantaviruses in Brazil.

## Discussion

The MCC composite tree (Appendix Figure) shows that all 38 samples from HPS patients and from rodents captured near human cases were related to ARAV or JUQV groups. Moreover, ARAV lineages obtained in the Central Plateau or southeastern region grouped in a robust monophyletic group independent of human or rodent origin. This finding reinforces the probability of zoonotic origin of human cases from a rodent-borne zoonotic pool and supports the view that closely related hantavirus lineages associated with distinct rodent species may be experiencing cross-species transmission (spillover) (*13**,**18*). ARAV appears to have dispersed from the cerrado region toward the southeast throughout São Paulo, as indicated by the basal position of the SS_DF_Nlas cluster in the MCC composite tree (Appendix Figure). JUQV dispersed across the southern temperate Araucaria pine forests and along the Atlantic rain forest, as shown by the basal position of SE_SC_Oni_1.

Several associations in the ARAV clade suggest movement of infected persons or dispersal of infected rodents. TB_SP_Hsp_18, which was isolated from a human case of HPS, was closely related to viruses from the Central Plateau. JU_SP_Hsp_11, ST_SP_Hsp_16, and AR_SP_Hsp_21 from the southeastern region were closely related to CO_GO_Hsp_20 from the Central Plateau. Data also indicated that genetic diversity of JUQV is greater than previously determined. SE_SC_Oni_1 from *O*. *nigripes* and AG_SP_Ost_1 from *O*. *stramineus* were basal taxa to JUQV with high support but may be the most divergent lineages so far detected. Levels of support in the MCC tree indicated that SG_MG_Nlas_8 and CX_RS_Hsp_22 would always branch from poorly defined nodes in the tree in a basal position relative to ARAV-Pergamino, JUQV, and Oran lineages. These results suggest that SG_MG_Nlas_8 and CX_RS_Hsp_22 may represent distinct lineages linking Argentinean Oran and Pergamino hantaviruses to lineages from which ARAV and JUQV originated.

Rooting of the tree and lineage associations suggest that ARAV and JUQV may have originated from a *Necromys* spp./*Akodon* spp.–associated hantavirus from the southern part of South America. This theory supports our finding of 1 *N*. *lasiurus* infected by ARAV (SG_MG_Nlas_9) and another *N*. *lasiurus* infected by a highly divergent lineage (SG_MG_Nlas_8) that outgroups with ARAV and JUQV. Both rodents were collected in São Gotardo in Minas Gerais State, at the edge of the central plains. The role of distinct rodent species as potential reservoirs and sources of human infection in Brazil and South America needs to be better understood. However, our data corroborate reports showing that *N*. *lasiurus* is a reservoir of ARAV, that *O*. *nigripes* is a reservoir of JUQV, and that rodents transmit hantaviruses to humans (*6**–**8*).

Rodent behavior is a factor in transmission of hantaviruses to humans, and *N*. *lasiurus* is an opportunistic and aggressive rodent species that is gradually being encroached upon in environments experiencing anthropogenic change in the southeastern region and the Central Plateau of Brazil. Conversely, *O*. *nigripes* has adapted to the Atlantic and Araucaria pine forests and has been found in lineal natural habitats bordering cultivated areas (*5**,**7**,**19*). Detection of AG_SP_Ost_1 in *O*. *stramineus* in the *O*. *nigripis*–associated JUQV group could be explained by virus spillover because *O*. *stramineus* is usually not infected with hantaviruses. Sampling also identified RP_SP_Ako_1 in an *Akodon* sp. rodent in Ribeirão Preto northwest of São Paulo State. This isolate branches in the tree within a well supported cluster of HPS cases reported in areas >50 miles of Ribeirão Preto. Distribution of *Akodon* spp. rodents includes the pampas grasslands of Argentina, and Bolivia, Paraguay, Uruguay, and southern Brazil. These rodents are known to be associated with hantaviruses in Argentina. Not yet determined is whether an *Akodon* sp. is transmitting hantavirus to humans in Ribeirão Preto (west of São Paulo State) and whether this infection was a recent cross-species transmission event from *N*. *lasiurus* to *Akodon* sp.

Samples we analyzed came from an extensive area that contained natural ecosystems largely degraded as a consequence of intensive agriculture and cattle raising. This region contains nearly one third of the population of Brazil in hundreds of towns and several large cities that grew during the 20th century. Other than agricultural expansion over a pristine environment, explosive and poorly planned urban expansion has also been responsible for degrading surrounding ecosystems. A recent estimate of the short-term rate of evolution of hantaviruses in South America indicates that divergence and spread of ARAV and JUQV is relatively recent, possibly within the past 200 years (*13*). Thus, increasing environmental and demographic changes during the past 100 years likely affected the ecology of wild rodent reservoirs and facilitated human infections and the emergence of HPS that we now observe.

Whether ARAV and JUQV differ in pathogenicity is unknown A possible distinction between these 2 groups is that the case-fatality rate in regions where ARAV has been isolated appears to be higher than that for JUQV. Higher case-fatality rates of HPS cases in regions with ARAV (Central Plateau and southeastern region) than in regions with JUQV (southeastern coast and southern region) suggest that ARAV is the most virulent hantavirus detected in Brazil (*6**,**7**,**19*). However, although ARAV has produced the most severe forms of HPS, many infections with ARAV are benign. This finding was observed in São Paulo State, where a serologic survey in Jardinópolis County showed that 14.3% of 32,000 local inhabitants had IgG antibodies to hantavirus (*20*), indicating that many persons were exposed to the hantavirus but did not have severe clinical symptoms. Further study is needed to determine which factors influence the severity of disease manifestation in humans caused by infections with hantaviruses, specifically with ARAV or JUQV (*6**–**8*).

We have identified the viruses circulating in our study area as ARAV and JUQV. Based on geographic distribution of these viruses and the assumption that no other unknown lineage is causing disease in humans, we suggest that ARAV may be responsible for >50% of HPS cases reported in Brazil. ARAV was associated with areas experiencing greater anthropogenic changes and disorganized human population growth than other more stable areas. ARAV may be the most virulent hantavirus in Brazil.

## Supplementary Material

Appendix Figure Maximum clade credibility (MCC) tree based on concatenation of partial sequences of nucleocapsid (N) and glycoprotein genes of hantavirus detected in 16 patients with hantavirus pulmonary syndrome and in 15 rodents, and other South American hantaviruses. Arrows toward the map show that main biomes in Brazil indicate that Araraquara hantavirus (ARAV) samples (shown in red) had a distribution ranging mainly from the Central Plateau (cerrado, a savanna-like ecosystem) to areas bordering the Atlantic rain forest in southeastern Brazil. Juquitiba hantavirus (JUQV) samples (shown in green) were found in the southern part of Brazil and were associated with Atlantic rain forest and subtropical Araucaria forest. Nucleocapsid gene sequences clustered either with ARAV 100% of the time in the 500 best maximum likelihood (ML) trees for the data and 88% of the time in 500 independent nonparametric bootstrap iterations or with JUQV 100% of the time in the 500 best ML trees for the data and 60% of the time in 500 independent nonparametric bootstrap iterations.
