# Supplementary figures and images for: Hantavirus Pulmonary Syndrome, Central Plateau, Southeastern, and Southern Brazil
Source: Emerg Infect Dis. 2009 Apr;15(4):561–7. doi: 10.3201/eid1504.080289 (PMC2671436; doi:10.3201/eid1504.080289)

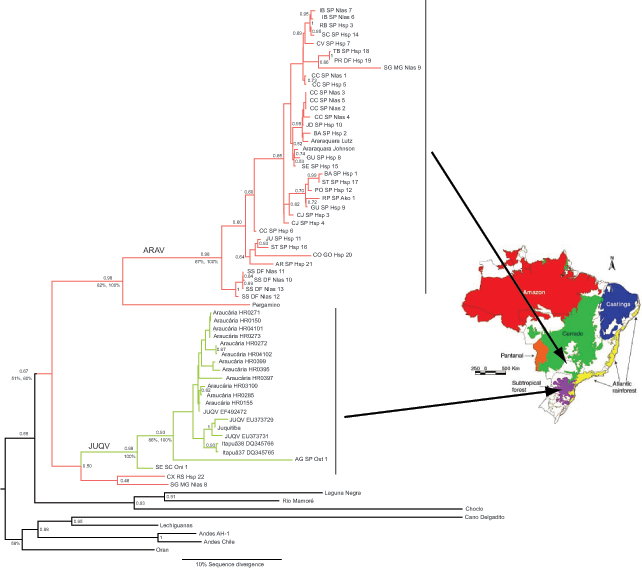

Supplement: Appendix Figure — Maximum clade credibility (MCC) tree based on concatenation of partial sequences of nucleocapsid (N) and glycoprotein genes of hantavirus detected in 16 patients with hantavirus pulmonary syndrome and in 15 rodents, and other South American hantaviruses. Arrows toward the map show that main biomes in Brazil indicate that Araraquara hantavirus (ARAV) samples (shown in red) had a distribution ranging mainly from the Central Plateau (cerrado, a savanna-like ecosystem) to areas bordering the Atlantic rain forest in southeastern Brazil. Juquitiba hantavirus (JUQV) samples (shown in green) were found in the southern part of Brazil and were associated with Atlantic rain forest and subtropical Araucaria forest. Nucleocapsid gene sequences clustered either with ARAV 100% of the time in the 500 best maximum likelihood (ML) trees for the data and 88% of the time in 500 independent nonparametric bootstrap iterations or with JUQV 100% of the time in the 500 best ML trees for the data and 60% of the time in 500 independent nonparametric bootstrap iterations. [file 08-0289_app-s1.gif]
